# HBK-15, a Multimodal Compound, Showed an Anxiolytic-Like Effect in Rats

**DOI:** 10.1007/s11064-022-03802-x

**Published:** 2022-11-09

**Authors:** Klaudia Lustyk, Kinga Sałaciak, Magdalena Jakubczyk, Magdalena Jastrzębska-Więsek, Anna Partyka, Anna Wesołowska, Henryk Marona, Karolina Pytka

**Affiliations:** 1 Department of Pharmacodynamics, Faculty of Pharmacy, Jagiellonian University Medical College, 9 Medyczna Street, 30-688 Krakow, Poland; 2 Department of Clinical Pharmacy, Faculty of Pharmacy, Jagiellonian University Medical College, 9 Medyczna Street, 30-688 Krakow, Poland; 3 Department of Bioorganic Chemistry, Chair of Organic Chemistry, Faculty of Pharmacy, Jagiellonian University Medical College, 9 Medyczna Street, 30-688 Krakow, Poland

**Keywords:** Anxiety, 2-Methoxyphenylpiperazine derivative, 5-HT_1A_ receptor, A rat model of anxiety, Radioligand binding

## Abstract

Anxiety is a common mental disorder, and its prevalence has lately increased because of the COVID-19 pandemic. Unfortunately, the available anxiolytics are often ineffective, and most possess addictive potential. Thus, searching for novel compounds is essential. In our previous studies, we selected a multimodal compound, HBK-15, which showed a fast antidepressant-like effect in animal models of depression. HBK-15 demonstrated a high affinity for serotonin 5-HT_1A_ receptors and moderate for 5-HT_7_, dopamine D_2_, and α_1_-adrenoceptors. Based on the receptor profile and preliminary studies, we aimed to investigate the anxiolytic potential of HBK-15 using the conditioned-response rat model of anxiety, i.e., the Vogel drinking test. We performed hot plate and free-drinking tests to exclude false positive results in the Vogel test. Using radioligand binding studies, we also investigated the affinity of the compound for the selected biological targets, which play a role in anxiety. Our experiments revealed that HBK-15 showed an anxiolytic-like effect in rats (5 mg/kg) without influencing the pain threshold or the amount of water consumed in the free-drinking test. Furthermore, the tested compound did not show a significant affinity for the selected biological targets, which suggests that its anxiolytic-like mechanism of action could be connected with the interaction with other receptors. This study indicates that multimodal compounds with a receptor profile similar to HBK-15 could be an attractive therapeutic option for patients with a generalized anxiety disorder. However, more studies are required to determine the exact mechanism of action of HBK-15 and its safety profile.

## Introduction

According to the World Health Organization, mental health conditions are increasing globally, affecting around 20% of the world’s young population [1, 2]. One of the most common mental disorders is anxiety, which can interfere with daily functioning causing not only constant fear, tension, irritability, restlessness, inattention, or insomnia, but also many serious physical symptoms such as tachycardia, increased blood pressure, trouble breathing, sweating, trembling or body pains [3]. Thus, patients suffering from anxiety disorders tend to have worse job performance, school productivity, problems in relationships, and overall decreased quality of life [4]. Moreover, the COVID-19 pandemic worsened this trend globally by increasing anxiety prevalence by 25% worldwide [5, 6].

Unfortunately, the available pharmacotherapy is often ineffective in treating anxiety disorder and only relieves symptoms or prevents panic attacks [7, 8]. In the past, the most prescribed anxiolytics were benzodiazepines - usually effective in improving symptoms but with a risk of serious side effects such as dependency, tolerance, somnolence, and memory impairments [9]. Subsequently, several selective serotonin reuptake inhibitors and serotonin-norepinephrine reuptake inhibitors (especially escitalopram and duloxetine) have been shown to reduce anxiety; however, their effects only appear after several weeks of treatment [10]. Another therapeutic option for patients with anxiety disorders is buspirone, which targets 5-HT_1A_ receptors. Nevertheless, the Cochrane review indicated its lower effectiveness than benzodiazepines or antidepressants [11]. Keeping in mind the ineffectiveness and limitations of anxiolytics, as well as the fact that in the last 5 to 10 years, much less research on novel anxiolytics was done in comparison to experimental treatments for depression, searching for novel compounds with higher efficacy and different mechanism of action is needed [8].

We have previously selected a novel 2-methoxyphenylpiperazine derivative, HBK-15. HBK-15 is a multimodal compound, showing a high affinity for serotonin 5-HT_1A_ receptors [12] and moderate towards serotonin 5-HT_7_ [12], dopamine D_2_ [13], and α_1_-adrenoceptors [14]. It also showed antagonistic properties at the 5-HT_3_ receptor in biofunctional assay [15]. Our studies demonstrated fast antidepressant-like effects of HBK-15 in mouse models of depression [13, 15]. Our preliminary study indicated the anxiolytic potential of HBK-15 [12]. Therefore, in this study, we aimed to investigate further the anxiolytic potential of HBK-15 using the conditioned-response rat model of anxiety, i.e., the Vogel drinking test.

## Materials and Methods

### Animals

In all experiments, we used male Wistar rats (200–220 g, in total 140 animals), purchased from the Animal House at the Faculty of Pharmacy, Jagiellonian University Medical College, Kraków, Poland. The animals were kept in groups of 3 rats in standard cages (42.5 × 26.5 × 18 cm) at constant room conditions (temperature: 22 ± 2 °C, humidity: 50 ± 10%). Behavioral experiments were performed between 8 am and 4 pm and evaluated by a trained observer blind to the treatments. Rats were handled for at least 3 days before starting the experimental procedures. Animals were randomly allocated to the treatment using a computer-generated sequence, and researchers making measurements on the animals or analyzing the results were blind to the allocation. All animals were used only once. Moreover, experimental groups were distributed across multiple cages, and the location of the cages in the room was changed following each day. All experimental procedures were approved by the Local Ethics Committee for Experiments on Animals in Kraków, Poland, and performed under the guidelines provided by the European Union Directive of 22 September 2010 (2010/63/EU) and Polish legislation concerning animal experimentation.

### Drugs

1-[(2-Chloro-6-methylphenoxy)ethoxyethyl]-4-(2-methoxyphenyl)piperazine hydrochloride (HBK-15) was synthesized in the Department of Bioorganic Chemistry, Chair of Organic Chemistry, Faculty of Pharmacy, Jagiellonian University Medical College [12]. The studied compound or diazepam (Sigma, Germany) was dissolved in saline and administered intraperitoneally (*ip*) in a 1 ml/kg volume. Control groups received saline. The doses of the studied compound for experiments were based on the earlier studies [16].

### In Vitro Experiments

#### Binding Assays

Binding studies were performed commercially in Eurofins Laboratories using testing procedures described elsewhere: melatonin 1 [17] and 2 [18], adenosine 1 [19], 2A [20], 2B [21] and 3 [22], neuropeptide Y 1 [23] and 2 [24], N neuronal α4β2 [25], N neuronal α7 [26], orexin 1 [27] and 2 [28], histamine 1 [29], 2 [30] and 3 [31], muscarinic 1 [32], 2 [32], and 3 receptors [33], and GABA transporter [34]. The results are presented as the inhibition of control-specific binding in the presence of HBK-15.

### In Vivo Experiments

#### Vogel Test

The testing procedure was based on a method of Vogel et al. [35] and used the Anxiety Monitoring System “Vogel test” produced by TSE Systems (Germany). It consisted of polycarbonate cages (dimensions 26.5 × 15 × 42 cm), equipped with a grid floor made from stainless steel bars and drinking bottles containing tap water. Experimental chambers were connected to PC software by control chassis and electric shocks generator. On the first day of the experiment, the rats were adapted to the test chambers and drank water from the bottle spout for 10 min. Afterward, the rats were returned to their home cages and were given 30 min free access to water, followed by a 24-h water deprivation period. The adaptation session and water deprivation protocols were repeated on the second day of the experiment. On the third day, the rats were placed again in the test chambers 30 min after HBK-15 or saline administration and given free access to the drinking tube. Recording data started immediately after the first lick, and rats were punished with an electric shock (0.5 mA, lasting 1 s) delivered to the metal drinking tube every 20 licks. The number of licks and the number of shocks received during a 5-min experimental session were recorded automatically. The Vogel conflict drinking test was employed as a “conditional” model where a noxious stimulus is applied.

#### Hot Plate and Free-Drinking Tests

To exclude possible drug-induced changes in shock sensitivity or an increasing influence on thirst drive, which can lead to false positive results in the Vogel conflict drinking test, stimulus threshold, and water consumption during a free-drinking session were determined in separate groups of rats. In either of those two studies, the rats were manipulated similarly to the Vogel conflict drinking test, including two 24-h water deprivation periods separated by 10-min adaptation session in experimental cages and 30-min of water availability in their home cages. In the free-drinking test, each animal was allowed to drink from the drinking bottle freely and the amount of water (g) consumed during 5 min was recorded for each rat. The pain threshold was evaluated using a hot plate test (Commat Ltd, Turkey) in rats. The plate was enclosed with a transparent Plexiglass cylinder (35 cm high) to keep the animal on the heated surface of the plate. The latency to pain reaction (lick a hind paw or jumping) when the rat was placed on a hot plate (52.5 ± 0.5 °C, 19-cm diameter) was measured. The rat was removed from the plate immediately upon visible pain reaction or if no response occurred within 30 s.

### Statistical Analysis

The number of animals in groups was based on our previous experiments [16]. Results are presented as means ± SD. Comparisons between experimental and control groups were performed by unpaired t-test or one-way ANOVA, followed by Dunnett’s *post hoc*. *p* < 0.05 was considered significant. All data were statistically evaluated with Prism 9.0 software (GraphPad Software, La Jolla, California, USA).

## Results

### HBK-15 Showed No Significant Affinity for the Selected Biological Targets

We investigated the affinity of HBK-15 for not yet tested selected receptors/transporters crucial for anxiolytic effect. The radioligand binding studies revealed that HBK-15 did not bind to any of the selected biological targets, i.e., melatonin 1 and 2, adenosine 1, 2A, 2B and 3, neuropeptide Y1 and 2, N neuronal α4β2, N neuronal α7, orexin 1 and 2, histamine 1, 2 and 3, muscarinic 1, 2 and 3 receptors, or GABA transporter (Table 1).

**Table 1:** In vitro binding assays for HBK-15

Molecular target	Source	% Inhibition of control specific binding
MT1	Human recombinant (CHO cells)	− 0.3
MT2	Human recombinant (CHO cells)	− 30.3
A1	Human recombinant (CHO cells)	− 0.7
A2A	Human recombinant (HEK-293 cells)	1.3
A2B	Human endogenous (HEK-293 cells)	− 3.0
A3	Human endogenous (HEK-293 cells)	16.8
Y1	Human endogenous (SK-N-MC cells)	0.2
Y2	Human endogenous (KAN-TS cells)	− 5.6
NTS1	Human recombinant (CHO cells)	2.9
N neuronal α4β2	Human recombinant (SH-SY5Y cells)	2.7
N neuronal α7	Human recombinant (SH-SY5Y cells)	10.3
OX1	Human recombinant (CHO cells)	− 13.2
OX2	Human recombinant (HEK-293 cells)	8.3
H1	Human recombinant (HEK-293 cells)	35.0
H2	Human recombinant (CHO cells)	9.2
H3	Human recombinant (CHO cells)	6.2
M1	Human recombinant (CHO cells)	27.1
M2	Human recombinant (CHO cells)	19.1
M3	Human recombinant (CHO cells)	5.6
GABA transporter	Wistar rat brain (minus cerebellum)	14.9

HBK-15 was tested at a concentration 10^−6^ M, except for MT1 and MT2 receptors, where the concentration used was 10^−7^ M. The results are presented as % inhibition of control specific binding. Results showing an activity > 50% were considered to represent significant effects of the test compound; results showing an inhibition between 25% and 50% indicates moderate to weak effect; results showing an inhibition < 25% are not considered significant and mostly attributable to the variability of the signal around the control level. Binding or functional studies were performed commercially in Eurofins Laboratories (Poitiers, France)

*MT* melatonin, *A* adenosine, *Y* neuropeptide, *NTS* neurotensin, *OX* orexin, *H* histamine, *M* muscarine

### HBK-15 Demonstrated an Anxiolytic-Like Effect in the Vogel Conflict Test

**Fig. 1:**
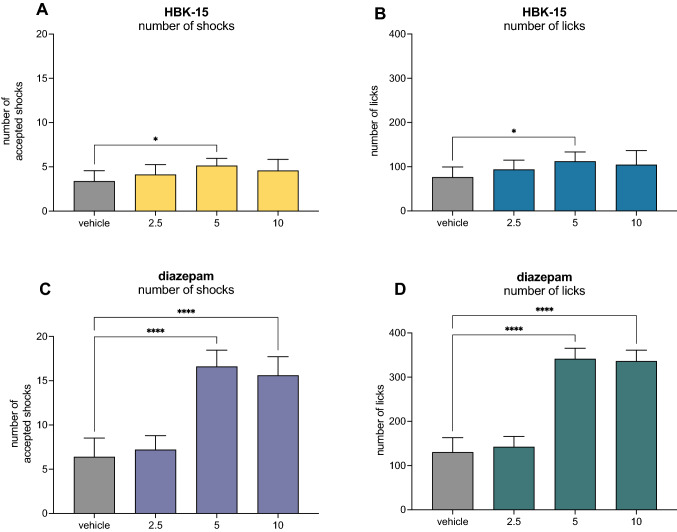
The effect of HBK-15 or diazepam on the number of shocks (**A**, **C**) and number of licks (**B**, **D**) in the Vogel conflict test. The test compound or vehicle (saline) were administered intraperitoneally (*ip*) 30 min before the test. The results are presented as bar plots showing the means ± SD. Statistical analysis: one-way ANOVA followed by Dunnett’s *post hoc* test, *p < 0.05, ****p < 0.0001; n = 7–8 rats per group

HBK-15 administered at a dose of 5 mg/kg increased the number of accepted shocks by 51.8% (*F*(3,27) = 3.5223, *p* < 0.05) and the number of licks by 46.8% (*F*(3,27) = 3.1472, *p* < 0.05) in the Vogel conflict test (Fig. 1).

Diazepam, used as a reference drug, administered at doses of 5 and 10 mg/kg (but not 2.5 mg/kg), produced an anti-conflict effect; it increased the number of accepted shocks by 160% and 143%, respectively (*F*(3,32) = 10.764, *p* < 0.0001) and the number of licks by 162% and 159%, respectively (*F*(3,32) = 11.466, *p* < 0.0001) in rats (Fig. 1).

### HBK-15 Did Not Affect the Animals’ Pain Reaction or Water Consumption

Neither HBK-15 at dose of 5 mg/kg nor diazepam at the doses of 5 and 10 mg/kg affected the pain reaction time in the hot plate test in rats (*t*(11) = 0.3144, ns and *F*(2,20) = 1.409, ns, respectively; Table 2). Similarly, none of the compounds changed the amount of liquid consumed by water-deprived rats during a 5-min session (*t*(10) = 0.07615, ns and *F*(2,22) = 0.239, ns, respectively; Table 2).

**Table 2:** The effect of HBK-15 in the hot plate and water consumption tests in water-deprived rats

Treatment	Dose (mg/kg)	Hot plate test Time of reaction [s]	Water consumption [g/5 min]
Vehicle	–	7.81 ± 1.81	5.49 ± 0.71
HBK-15	5	7.43 ± 2.55	5.45 ± 1.13
Vehicle	0	8.20 ± 2.38	5.30 ± 0.79
Diazepam	5	11.40 ± 4.76	5.30 ± 1.06
	10	10.06 ± 3.70	5.00 ± 0.79

HBK-15, diazepam, or vehicle (saline) were injected intraperitoneally (*ip*) 30 min before the test. The results are presented as means ± SD of time reaction in the hot plate test and amount of water consumed during 5-min test session. Statistical analysis: unpaired t test or one-way ANOVA followed by Dunnett’s *post hoc* test, n = 6–9 rats per group

## Discussion

We found that HBK-15 showed an anxiolytic-like effect in the Vogel’s test in rats. The lowest effective dose in this test was the same as for diazepam, an anxiolytic drug. The compound did not bind to the selected biological targets, suggesting that its effects might be mediated via other receptors/transporters, such as the 5-HT_1A_, or 5-HT_7_ receptors.

Many receptors and transporters play a role in anxiety [36–39]. Most drugs in the clinic target the GABAergic system, but research has shown that not only GABA receptors are involved in the pathomechanisms of anxiety. Scientists indicated an important role of serotonin, dopamine, adenosine, or nicotinic preceptors [36–39]. Our previous experiments showed that HBK-15 has a high affinity for serotonin 5-HT_1A_ and moderate for 5-HT_7_, dopamine D_2_, or α_1_-adrenoceptors [14–16, 40]. Knowing that HBK-15 targets several receptors, as the first step, we investigated whether the compound influences other biological targets, which could be important for anxiolytic effects, i.e., melatonin 1 and 2 receptors, adenosine 1, 2A, 2B, and 3 receptors, neuropeptide Y 1 and 2 receptors, N neuronal α4β2 and N neuronal α7 receptors, orexin 1 and 2 receptors, histamine 1, 2 and 3 receptors, muscarinic 1, 2, and 3 receptors, and GABA transporter. The radioligand binding studies showed that HBK-15 did not bind significantly with either of the studied biological targets. In our previous studies, HBK-15 showed a high affinity for the 5-HT_1A_ receptor (*p*Ki = 9 [12], and moderate for serotonin 5-HT_7_ (*p*Ki = 7,47 [12]), dopamine D_2_ (*p*Ki = 7,27 [13]), and α_1_-adrenoceptors (*p*Ki = 7,89 [14]). The compound also showed antagonistic properties at the 5-HT_3_ receptor in the bifunctional assay (*p*K_B _= 7361 [15]). However, its affinity for the 5-HT_3_ receptor is yet to be tested. Moreover, HBK-15 did not show a significant affinity for GABA_A_ receptor [40]. Thus, the observed pharmacological effect of the compound is most likely due to the interaction with either the above receptors or other not-yet-tested biological targets.

Interestingly, HBK-15 showed no significant affinity for histamine or muscarinic receptors, which agrees with our previous biofunctional studies [41, 42]. Affinity for histamine or muscarinic receptors is an undesirable feature of central-acting compounds, as interaction with these receptors may cause side effects such as weight gain, sedation, tachycardia, blurred vision, and others [43, 44]. Thus, the obtained results encourage further studies on HBK-15.

As the next step, we investigated the potential anxiolytic properties of HBK-15 using a punishment-induced conflict test in rats, i.e., the Vogel conflict test. The test predicts drugs that can effectively treat generalized anxiety disorders and acute anxiety states [45]. Vogel test is based on the approach-avoidance conflict generated in rodents between an appetitive drive: to drink water after a period of water deprivation and the fear of doing so as water consumption is punished by electric shocks delivered either to the animal’s paws or tongue [46]. HBK-15 showed an anxiolytic-like effect in the Vogel conflict test. Since the compound did not affect pain threshold or water consumption, the observed effect is specific to the anxiolytic-like effect.

Interestingly, we observed an inverted U-shaped effect for HBK-15 (only 5 mg/kg dose was effective). This common effect is observed in neuropharmacology and is not fully understood [47–50]. However, in the case of HBK-15, it might be related to its effect on several receptors, i.e., depending on the dose, we observe a different level of activation of receptors that HBK-15 targets. On the other hand, at higher doses, the sedative effect of HBK-15 [12] might mask the anxiolytic-like effect. Nevertheless, explaining this issue requires further studies.

Finally, it is worth mentioning that HBK-15 showed an anxiolytic-like effect at the same dose as diazepam, a drug with proven anxiolytic properties. However, in contrast with HBK-15, diazepam showed its anxiolytic properties also at a two-fold higher dose (10 mg/kg). The obtained results agree with our previous experiments showing that anxiolytic-like properties of HBK-15 in rats in the ethological conflict test – the elevated plus maze or animal models of depression in mice [13, 15, 16]. Together these findings strongly suggest that compounds with a receptor profile like HBK-15 might have potential in the treatment of anxiety disorders.

The study has some limitations. First, we assessed the pharmacological effects using the Vogel test in rats only after a single administration. In generalized anxiety, anxiolytics should be taken daily; thus, investigating the effects of HBK-15 after chronic administration is necessary. Next, in future studies, we should test which receptors targeted by HBK-15 are predominantly engaged in the anxiolytic-like effect of the compound. Such information would make it possible to target the synthesis of new, more effective compounds with anxiolytic properties and potential use in the treatment of generalized anxiety.

## Conclusion

Our study suggests that multimodal compounds with a receptor profile like HBK-15, i.e., targeting 5-HT_1A_ and, to a lesser extent, 5-HT_7_ and D_2_ receptors, could be attractive therapeutic option for patients with generalized anxiety disorder. However, more studies are required to determine the exact mechanism of action of HBK-15 and its safety profile.
